# Smaller pineal gland is associated with rapid eye movement sleep behavior disorder in Alzheimer’s disease

**DOI:** 10.1186/s13195-020-00725-z

**Published:** 2020-11-21

**Authors:** Jeongbin Park, Seung Wan Suh, Grace Eun Kim, Subin Lee, Jun Sung Kim, Hye Sung Kim, Seonjeong Byun, Jong Bin Bae, Jae Hyoung Kim, Sang Eun Kim, Ji Won Han, Ki Woong Kim

**Affiliations:** 1 Department of Brain and Cognitive Sciences, Seoul National University College of Natural Sciences, Seoul, Korea; 2 Department of Psychiatry, Kangdong Sacred Heart Hospital, Hallym University College of Medicine, Seoul, Korea; 3 Department of Neuropsychiatry, Seoul National University Bundang Hospital, Seongnam, Korea; 4 Department of Radiology, Seoul National University Bundang Hospital, Seongnam, Korea; 5 Department of Radiology, Seoul National University College of Medicine, Seoul, Korea; 6 Department of Nuclear Medicine, Seoul National University Bundang Hospital, Seongnam, Korea; 7 Department of Nuclear Medicine, Seoul National University College of Medicine, Seoul, Korea; 8 Department of Psychiatry, Seoul National University College of Medicine, Seoul, Korea

**Keywords:** Alzheimer’s disease, Pineal gland, Rapid eye movement sleep behavior disorder, Magnetic resonance imaging, Amyloid positron emission tomography

## Abstract

**Background:**

To investigate the association between pineal gland volume and symptoms of rapid eye movement (REM) sleep behavior disorder (RBD) in Alzheimer’s disease (AD) patients without any feature of dementia with Lewy bodies.

**Methods:**

We enrolled 296 community-dwelling probable AD patients who did not meet the diagnostic criteria for possible or probable dementia with Lewy bodies. Among them, 93 were amyloid beta (Aβ) positive on ^18^F-florbetaben amyloid brain positron emission tomography. We measured RBD symptoms using the REM Sleep Behavior Disorder Screening Questionnaire (RBDSQ) and defined probable RBD (pRBD) as the RBDSQ of 5 or higher. We manually segmented pineal gland on 3T structural T1-weighted brain magnetic resonance imaging.

**Results:**

The participants with pRBD had smaller pineal parenchyma volume (VPP) than those without pRBD (*p* <  0.001). The smaller the VPP, the more severe the RBD symptoms (*p* <  0.001). VPP was inversely associated with risk of prevalent pRBD (odds ratio = 0.909, 95% confidence interval [CI] = 0.878–0.942, *p* <  0.001). Area under the receiver operator characteristic curve for pRBD of VPP was 0.80 (95% CI = 0.750–0.844, *p* <  0.0001). These results were not changed when we analyzed the 93 participants with Aβ-positive AD separately.

**Conclusions:**

In AD patients, reduced pineal gland volume may be associated with RBD.

## Background

Rapid eye movement (REM) sleep behavior disorder (RBD) is a parasomnia characterized by loss of normal skeletal muscle atonia accompanied by dream-enacting behaviors [1]. A large autopsy study found that 94% of the neurodegenerative disorders associated with RBD were synucleinopathies and claimed that the presence of RBD should at least raise suspicion of primary or coexisting Lewy body disease even in the typical Alzheimer’s disease (AD) [2]. However, their claim seems somewhat overextended. First of all, their study sample may not represent overall RBD and be subject to a sampling bias [2]. Second, RBD is also prevalent in cognitively normal older adults [3], suggesting that RBD may occur without synucleinopathy. AD is far more prevalent than synucleinopathies [4], and RBD was quite common in numerous cross-sectional and prospective studies on AD patients [5–15]. There is no reason to assume that AD patients will develop RBD only from synucleinopathy, not the pathologies that can lead to RBD in normal older adults without synucleinopathy.

In cognitively normal older adults, the smaller pineal gland was associated with more RBD symptoms and higher risk of incident RBD symptoms, suggesting that reduction of melatonin secretion associated with the reduction of pineal gland volume may be a potential cause of RBD [16]. AD patients show reduced endogenous melatonin levels [17] and have a smaller pineal gland compared to healthy controls [18]. Pineal gland is a small neuroendocrine organ, and its primary function is to regulate sleep through the synthesis and secretion of melatonin [19]. In humans, roughly 80% of the pineal gland comprises melatonin-producing pinealocytes [19], and pineal gland volume is proportional to the endogenous melatonin levels [20, 21]. Pineal gland volume can be changed by various physiological or pathological conditions that may change melatonin production [16, 22]. In a couple of clinical trials, RBD symptoms such as dream-enacting behaviors and REM sleep muscle atonia were improved by the administration of melatonin [23, 24] but relapsed by its discontinuation [23]. However, the association between pineal gland and RBD has never been investigated in AD patients.

In this study, we investigated the association between pineal gland volume and RBD symptoms in probable AD patients who did not meet the diagnostic criteria of possible and probable dementia with Lewy bodies (DLB) [25].

## Methods

### Participants

We enrolled 296 community-dwelling probable AD from the visitors to the Dementia Clinic of the Seoul National University Bundang Hospital (SNUBH) from 2011 to 2020. Among them, 104 underwent a ^18^F-florbetaben amyloid brain positron emission tomography (PET) scan, and 93 were found to be amyloid beta (Aβ)-positive.

We excluded the participants with following conditions: possible or probable DLB or Parkinson’s disease dementia (PDD); any major psychiatric and/or neurological disorders that could affect cognitive function other than AD; any history of brain tumors, substance abuse or dependence, and use of medications such as clonazepam or exogenous melatonin that may influence RBD symptom; any serious medical conditions that could affect the structure and/or function of the pineal gland or abnormalities in pineal gland morphology such as neoplastic lesions or extremely large cystic gland (diameter greater than 15.0 mm) [26]; and those with high risk of restless legs syndrome (positive on Cambridge-Hopkins Restless Legs Syndrome questionnaire) [27] and obstructive sleep apnea (STOP-BANG questionnaire score of ≥ 5 points) [28], all of which could mimic symptoms of RBD [29, 30].

All participants were fully informed with the protocol of this study, and provided written informed consents signed by themselves or their legal guardians. This study was approved by the Institutional Review Board of the SNUBH.

### Diagnostic assessments

Geriatric psychiatrists with expertise in dementia research conducted in person standardized diagnostic interviews, detailed medical histories, and physical/neurological examinations using the Korean version of the Consortium to Establish a Registry for Alzheimer’s Disease Assessment Packet Clinical Assessment Battery (CERAD-K) [31] and the Korean version of the Mini-International Neuropsychiatric Interview [32]. Additionally, research neuropsychologists administered the CERAD-K Neuropsychological Assessment Battery (CERAD-K-N) [31, 33], Digit Span Test [34], Frontal Assessment Battery [35], and Geriatric Depression Scale [36].

Trained research nurses collected data on age, sex, years of education, duration of AD (months), intracranial volume (ICV), history of head injury, amount of smoking (packs/day) and alcohol drinking (standard units/week) over the past 12-month period, and use of drugs influencing sleep or motor activity, including cholinesterase inhibitors (donepezil, rivastigmine, and galantamine), antidepressants (selective serotonin reuptake inhibitor, serotonin norepinephrine reuptake inhibitor, and others), carbamazepine, triazolam, zopiclone, quetiapine, clozapine, and sodium oxybate to each participant. We diagnosed dementia according to the fourth edition of the Diagnostic and Statistical Manual of Mental Disorders Text Revision criteria [37]. Global severity of dementia was determined according to the Clinical Dementia Rating [38]. We determined probable AD according to the National Institute of Neurological and Communicative Disorders and Stroke/Alzheimer’s Disease and Related Disorders Association diagnostic criteria [39]. We diagnosed probable or possible DLB and PDD according to the diagnostic criteria proposed by McKeith et al. [25], in which the presence of RBD features was ignored in the current study.

### Assessment of brain amyloid deposition

We performed ^18^F-florbetaben amyloid brain PET imaging using a Discovery VCT scanner (General Electric Medical Systems; Milwaukee, WI, USA) in three-dimensional (3D) acquisition mode. The participants were injected with 8.1 mCi (300 MBq) of ^18^F-florbetaben (Neuraceq) as a slow single intravenous bolus (6 s/mL) in a total volume of up to 10 mL. After a 90-min uptake period, we obtained 20-min PET images comprising four 5-min dynamic frames. The determination was based on the visual interpretation of tracer uptake in the gray matter of the following four brain regions: the temporal lobes, frontal lobes, posterior cingulate cortex/precuneus, and parietal lobes. Participants were considered Aβ positive if smaller areas of tracer uptake were equal to or higher than those present in the white matter extending beyond the white matter rim to the outer cortical margin involving the majority of the slices within at least one of the four brain regions (“moderate” Aβ deposition) or a large confluent area of tracer uptake (i.e., signal intensity) was equal to or higher than that present in the white matter extending beyond the white matter rim to the outer cortical margin and involving the entire region including the majority of slices within at least one of the four brain regions (“pronounced” Aβ deposition). Participants were considered Aβ negative if tracer uptake in the gray matter is lower than that in the white matter in all four brain regions (no β-amyloid deposition).

### Assessment of rapid eye movement sleep behavior disorder symptoms

We evaluated behavioral features of RBD using the REM Sleep Behavior Disorder Screening Questionnaire (RBDSQ) [40]. The RBDSQ is a self-reported screening instrument used to diagnose RBD and comprises 10 items assessing the most prominent clinical features of RBD: items 1 to 4, the frequency and content of dreams and their relationship to nocturnal movements and behaviors; item 5, self-injuries and injuries to the bed partner; item 6, four subsections specifically assessing nocturnal motor behavior (e.g., questions about nocturnal vocalization (6.1), sudden limb movements (6.2), complex movements (6.3), or bedside items that fall down (6.4)); items 7 and 8, nocturnal awakenings; item 9, disturbed sleep in general; and item 10, the presence of any neurological disorder. Each item could be answered as “yes” or “no.” The RBDSQ score ranges from 0 to 13 points, with higher scores indicating more features associated with RBD. We defined probable RBD (pRBD) as having a total score of 5 or higher on the RBDSQ [40]. The questionnaire was completed by the participants with the corroboration from their partners.

### Assessment of pineal gland volume

**Fig. 1:**
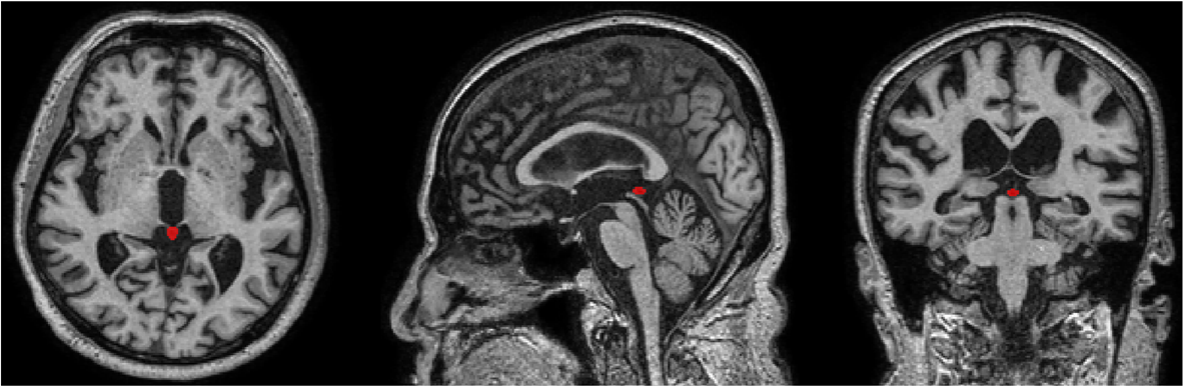
Assessment of pineal gland volume on 3D T1-weighted brain magnetic resonance images at 1.0 × 1.0 × 1.0 mm^3^. The pineal gland was manually segmented from surrounding cerebrospinal fluid space

We obtained 3D structural T1-weighted spoiled gradient echo magnetic resonance (MR) images using a Philips 3.0 Tesla Achieva scanner (Philips Medical Systems; Eindhoven, the Netherlands) within 3 months of clinical assessments with the following parameters: acquisition voxel size = 1.0 × 0.5 × 0.5 mm; sagittal slice thickness = 1.0 mm; repetition time = 4.61 ms; echo time = 8.15 ms; number of excitations = 1; flip angle = 8°; field of view = 240 × 240 mm; and acquisition matrix size = 175 × 256 × 256 mm in the *x*-, *y*-, and *z*-dimensions. We implemented bias field correction to remove the signal intensity inhomogeneity artifacts of MR images using Statistical Parametric Mapping software (version 12, SPM12; Wellcome Trust Centre for Neuroimaging, London; http://www.fil.ion.ucl.ac.uk/spm). We resliced the MR images into an isotropic voxel size of 1.0 × 1.0 × 1.0 mm^3^. We measured ICV using FreeSurfer software (version 5.3.0; http://surfer.nmr.mgh.harvard.edu) to adjust for interindividual variabilities in brain volume. We assessed pineal gland volume as described in our previous work [16]. In brief, trained researchers constructed a 3D mask of each pineal gland by manually segmenting the pineal gland slice-by-slice on the resliced T1-weighted MR images at 1.0 × 1.0 × 1.0 mm^3^ using the ITK-SNAP software (version 3.4.0; http://www.itksnap.org). We measured pineal gland volume and pineal cysts volume and estimated the volume of pineal parenchyma (VPP) by subtracting the pineal cysts volume from the pineal gland volume (Fig. 1). We defined a pineal cyst as an area of homogenous intensity that was isointense to the cerebrospinal fluid in T1 sequence images with a diameter of 2.0 mm or greater [41].

The intra-rater and inter-rater intraclass correlation coefficient were 0.983 (95% confidence interval [CI] = 0.956–0.993, *p* <  0.001) and 0.934 (CI = 0.828–0.974, *p* <  0.001), respectively.

### Statistical analyses

We compared the continuous variables using the independent samples *t* tests and categorical variables using the chi-squared tests between groups. We compared VPP between the participants with pRBD and those without pRBD using analysis of covariance that adjusted for age, sex, years of education, ICV, head injury, smoking, alcohol drinking, and use of drugs influencing sleep or motor activity as covariates. We examined the association between VPP and the risk of pRBD using binary logistic regression analysis that was adjusted for the same covariates. We examined the diagnostic performance of the VPP for pRBD using the receiver operating characteristic (ROC) analysis. We calculated the optimal cutoff value and area under the curve (AUC) using Youden index maximum (sensitivity + specificity − 1). We examined the association of VPP with RBDSQ total score (RBDSQ-T) and the item-6 score of the RBDSQ (RBDSQ-6) using multiple linear regression model adjusted for the covariates stated above. The RBDSQ item 6 comprises four subitems on the core symptoms of RBD: nocturnal vocalization (6.1), sudden limb movements (6.2), complex movements (6.3), or bedside items that fall down (6.4). We conducted the same analyses only on the Aβ-positive AD patients.

For all analyses, we considered a two-tailed *p* value less than 0.05 as statistically significant and employed Bonferroni corrections to reduce type I error when multiple comparisons were conducted. We performed ROC analyses using MedCalc for Windows version 18.11.3 (MedCalc Software, Mariakerke, Belgium) and all the other statistical analyses using the Statistical Package for the Social Sciences for Windows version 20.0 (International Business Machines Corporation, Armonk, NY).

## Results

**Fig. 2:**
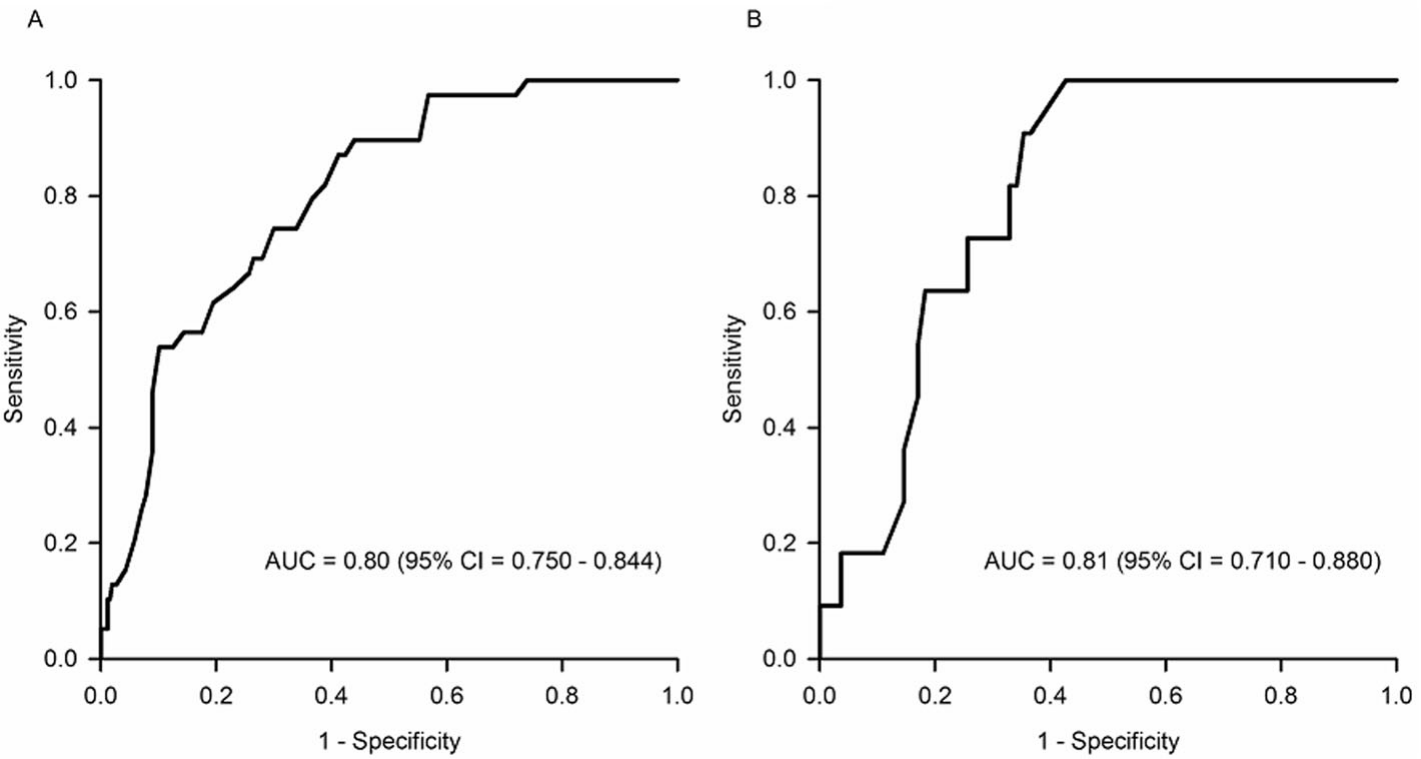
Diagnostic accuracy for the prevalent probable rapid eye movement sleep behavior disorder of the pineal parenchyma volume in **a** all participants and **b** participants with Aβ-positive Alzheimer’s disease. Aβ, amyloid beta; VPP, pineal parenchyma volume (mm^3^); AUC, area under the curve; CI, confidence interval

**Fig. 3:**
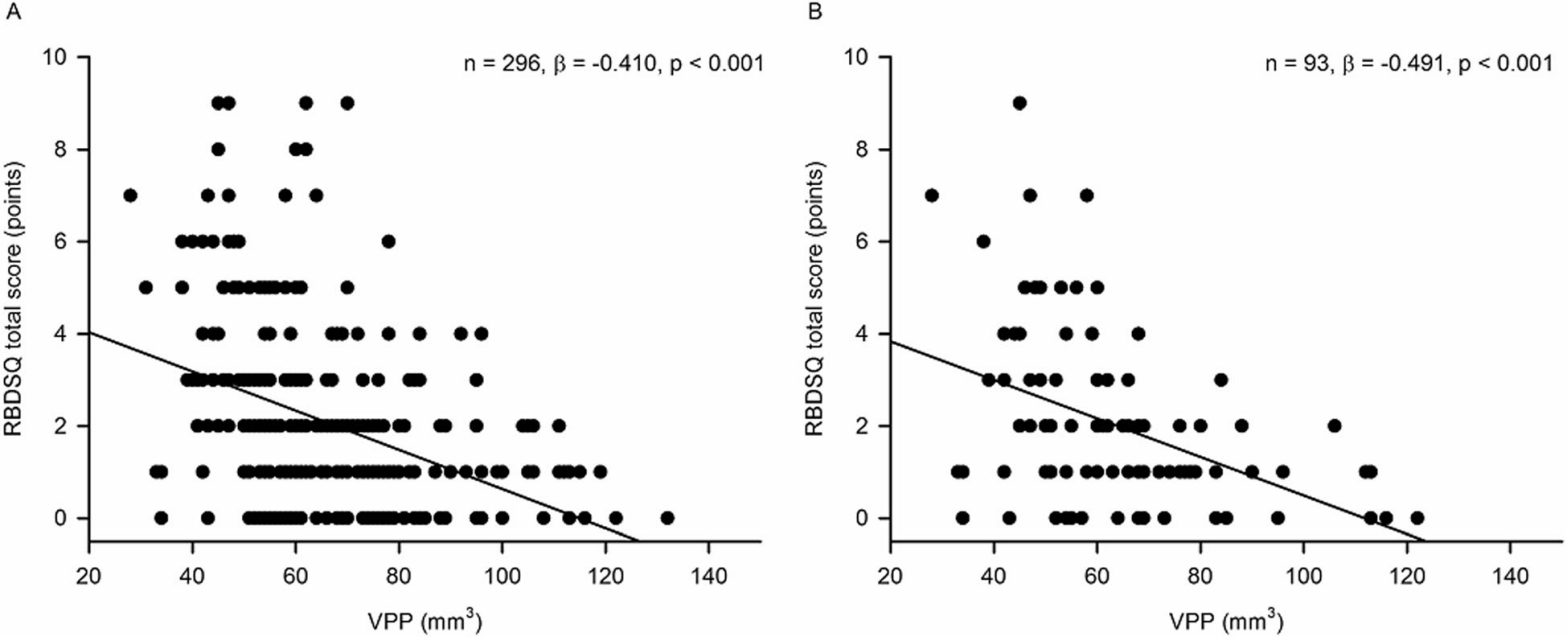
Association between REM Sleep Behavior Disorder Screening Questionnaire total score and pineal parenchyma volume (mm^3^) in **a** all participants and **b** participants with Aβ-positive Alzheimer’s disease. Multiple linear regression model adjusted for age, sex, years of education, intracranial volume, head injury, amount of smoking, amount of alcohol drinking, and use of drugs influencing sleep or motor activity

As summarized in Table 1, the AD patients with pRBD showed smaller VPP than those without pRBD (*p* < 0.001). VPP was inversely associated with the risk of pRBD (odds ratio [OR] = 0.909, 95% CI = 0.878–0.942, *p* < 0.001), indicating that the AD patients with smaller VPP may be more likely to have pRBD than those with smaller VPP. The AUC of VPP for pRBD was 0.80 (95% CI = 0.750–0.844, *p* < 0.0001, Fig. 2a), and the optimal cutoff value for classifying pRBD was 62 mm^3^ (sensitivity = 87.18%; specificity = 58.75%). VPP was also inversely associated with the RBDSQ-T (standardized *β* = − 0.410, *p* < 0.001) and the RBDSQ-6 (standardized *β* = − 0.224, *p* < 0.001, Fig. 3a).

**Table 1:** Demographic and clinical characteristics of the participants

	Without pRBD (*n* = 257)	With pRBD (*n* = 39)	*p*
Age (years, mean ± SD)	77.4 ± 7.4	76.8 ± 7.4	0.634^a^
Sex (women, %)	69.3	79.5	0.191^a^
Education (years, mean ± SD)	9.9 ± 5.6	8.1 ± 5.5	0.065^a^
Presence of cohabitants, (present, %)	80.5	74.4	0.371^a^
Duration of AD (months, mean ± SD)	36.9 ± 25.7	44.0 ± 37.8	0.265^a^
Drugs influencing sleep or motor activity (users, %)	29.2	38.5	0.240^a^
History of head injury (present, %)	9.0	10.3	0.792^a^
Alcohol drinking (SU/week, mean ± SD)	1.8 ± 7.1	0.7 ± 3.4	0.375^a^
Smoking (packs/day, mean ± SD)	0.1 ± 0.6	0.0 ± 0.2	0.750^a^
GDS (points, mean ± SD)	12.2 ± 6.9	16.5 ± 6.7	< 0.001^a^
CDR (points, mean ± SD)	0.7 ± 0.4	0.9 ± 0.5	0.903^a^
STOP-BANG (points, mean ± SD)	2.3 ± 0.9	2.6 ± 1.0	0.041^a^
RBDSQ (points, mean ± SD)			
Total score	1.4 ± 1.2	6.1 ± 1.4	< 0.001^a^
Item-6 score	0.2 ± 0.5	1.2 ± 1.2	< 0.001^a^
Intracranial volume (cm^3^, mean ± SD)	1515.5 ± 147.7	1509.1 ± 154.1	0.805^a^
VPP (mm^3^, mean ± SD)	69.5 ± 18.5	51.7 ± 10.8	< 0.001^b^
Cerebral amyloid deposition (present, %)	31.9	28.2	0.643^a^

*Abbreviations*: *pRBD* probable REM sleep behavior disorder, *SD* standard deviation, *AD* Alzheimer’s disease, *SU* standard units, *GDS* Geriatric Depression Scale, *CDR* Clinical Dementia Rating, *RBDSQ* REM Sleep Behavior Disorder Screening Questionnaire, *VPP* pineal parenchyma volume

^a^Independent sample *t* test for continuous variables and chi-square test for categorical variables

^b^Analysis of covariance adjusted for age, sex, years of education, intracranial volume, head injury, amount of smoking, amount of alcohol drinking, and use of drugs influencing sleep or motor activity

These results were not changed when we analyzed the Aβ-positive AD patients separately. Among the 93 participants with Aβ-positive AD, 11 (11.83%) had pRBD. The Aβ-positive AD patients with pRBD showed smaller VPP than those without pRBD (*p* = 0.002). VPP was inversely associated with the risk of pRBD (OR = 0.901, 95% CI = 0.840–0.966, *p* = 0.004), and AUC of VPP for pRBD was 0.81 (95% CI = 0.710–0.880, *p* < 0.0001; Fig. 2b). The optimal cutoff value of the VPP for classifying pRBD was 60 mm^3^ (sensitivity = 100%; specificity = 57.32%). VPP also showed significant inverse association with the RBDSQ-T (standardized *β* = − 0.491, *p* < 0.001) and the RBDSQ-6 (standardized *β* = − 0.276, *p* = 0.015, Fig. 3b).

## Discussion

In this cross-sectional study, we found that smaller pineal parenchyma volume was associated with more RBD symptoms in AD patients, which is in line with our previous observation that smaller pineal parenchyma volume was associated with the more RBD symptoms and the higher risk of future pRBD in cognitively normal older adults [16].

It is now well established that RBD is a strong predictor of neurodegeneration, in particular α-synucleinopathies [1]. According to a previous clinicopathological study, 94% of the polysomnography (PSG)-confirmed RBD patients were found to have synucleinopathies at autopsy [2], suggesting that the presence of RBD in patients with dementia may favor the diagnosis of DLB [42]. However, not all RBD patients progressed to neurodegenerative syndrome with synucleinopathies. The overall conversion rate from idiopathic RBD to an overt neurodegenerative syndrome was 6.3% per year in the elderly adults aged 66.3 ± 8.4 years on average [43]. Furthermore¸ RBD can occur alone without any neurological conditions, and large clinical series have reported that the idiopathic form of RBD accounts for up to 60% of the cases [3]. Therefore, we should be more cautious in confirming that all dementia with RBD is a synucleinopathy or at least a neurodegenerative disease having synucleinopathies as a secondary pathology. Although synucleinopathies may be a common sufficient condition for RBD, it is not a necessary condition for RBD.

RBD was common in clinically diagnosed AD [5, 6] and 3–11% of polysomnography-defined RBD patients developed AD [9–14]. In amyloid PET-confirmed AD patients, 24.6% showed RBD in a previous study [8], and 11.8% showed pRBD in the current study. Some authors have argued that an imbalance of acetylcholine transmission, a hallmark of AD, could explain the occurrence of RBD in a small portion of AD patients [6]. This is based on the findings that acetylcholine may be involved in the induction of REM sleep atonia [15], considering that an injection of cholinergic agonists induced muscle atonia in dogs [44] and the administration of cholinesterase inhibitors augmented the amount of REM sleep [45]. The brainstem regions also have been implicated in RBD pathophysiology based on lesion studies in animals, especially involving pontine nuclei including the noradrenergic locus coeruleus (LC), cholinergic pedunculopontine nucleus, and laterodorsal tegmental nucleus [1]. Lesioning the LC causes REM sleep without atonia, and size of the lesion determines whether simple or complex behaviors are exhibited [46]. The LC is prone to early neurodegeneration [47], and LC neurons can be lost up to 70% in AD brains [48]. Therefore, atrophy of LC nuclei with impaired noradrenergic systems may also contribute to the development of RBD in AD patients [6].

In our previous and the current works, we demonstrated the association of smaller pineal gland with the risk of pRBD in both cognitively normal older adults without any symptom or sign of neurodegenerative disorders including synucleinopathies [16] and in AD patients without any symptom or sign of synucleinopathies. These results suggested that reduced endogenous melatonin production may be another cause of RBD in AD patients as well as in normal older adults because the secretion of melatonin was strongly associated with pineal gland volume. Compared to healthy controls, AD patients showed disrupted circadian melatonin rhythm, lower melatonin levels in the cerebrospinal fluid, serum and postmortem pineal gland [17], and smaller pineal parenchyma [18]. Since the pineal gland is a circumventricular organ surrounded by the cerebrospinal fluid [19], it can be easily influenced by soluble Aβ peptides [49]. A previous in vitro study of isolated rat pineal glands confirmed that Aβ directly inhibited pineal melatonin synthesis and impaired melatonergic systems, leading to a neuroinflammatory response within the gland [49]. Therefore, enduring insults of Aβ may reduce pineal gland volume and melatonin production, which may increase the risk of RBD in AD patients. In addition, under physiological conditions, melatonin in vivo protects central cholinergic neurons against Aβ-mediated toxicity via its antioxidant and anti-amyloidogenic properties [50]. Melatonin not only inhibits Aβ generation but also arrests the formation of amyloid fibrils by a structure-dependent interaction with Aβ [50]. Therefore, reduced melatonin production due to pineal atrophy may also increase the risk of RBD or worsen RBD symptoms in AD patients indirectly via reduced protection of the cholinergic system from amyloid toxicity.

## Limitations

Our study has several methodological limitations. First, we used a questionnaire to determine if a participant was at a high risk of RBD, whereas video PSG is required to establish the definitive diagnosis of RBD [1]. This could be a substantial problem when the participants have significant cognitive impairments such as AD, leading to a recall bias. However, considering that the previous reports have suggested that the prevalence of PSG-confirmed RBD in AD subjects ranges from 5% (mean age [SD], 70.5 [9.4]; mean disease duration of AD, 16.1 [7.1] months) [6] to 27% (mean age, 70.2 [5.6] with global deterioration scale score of 3 or 4 [5], our results, with the prevalence of pRBD of 13% (mean age, 77.3 [7.4]; mean disease duration of AD, 37.8 [27.6] months), seem to be in a reasonable extent. Additionally, we obtained the RBDSQ data with the corroboration from the participant’s partners, which could increase their validity. Second, although we strictly excluded AD patients who simultaneously met the diagnostic criteria for possible or probable DLB, it is still possible that our study samples could have included the patients with synucleinopathies because clinical features between AD and DLB are overlapping [51] and 40–50% of AD patients had α-synuclein-positive Lewy bodies [52–54]. In addition, we did not conduct brain dopamine transporter scan or metaiodobenzylguanidine myocardial scan which would have helped to rule out DLB more definitively. However, even in synucleinopathies, the pineal gland may be associated with the risk of RBD because melatonin also played a protective role against synucleinopathies [55]. Third, causal relationship between pineal gland volume and pRBD cannot be inferred because the current study employed a cross-sectional design.

## Conclusion

In conclusion, the current study suggests that smaller pineal gland may be associated with the risk and/or severity of RBD in AD patients.

## Data Availability

The datasets used/or analyzed during the current study are available from the corresponding author on reasonable request.

## Abbreviations

AD: Alzheimer’s disease
AUC: Area under the curve
Aβ: Amyloid beta
CERAD: Consortium to Establish a Registry for Alzheimer’s Disease
CI: Confidence interval
DLB: Dementia with Lewy bodies
ICC: Intraclass correlation coefficient
ICV: Intracranial volume
LC: Locus coeruleus
MRI: Magnetic resonance imaging
OR: Odds ratio
PDD: Parkinson’s disease dementia
PET: Positron emission tomography
pRBD: Probable rapid eye movement sleep behavior disorder
PSG: Polysomnography
RBD: Rapid eye movement sleep behavior disorder
RBDSQ: Rapid Eye Movement Sleep Behavior Disorder Screening Questionnaire
RBDSQ-6: Item-6 score of the RBDSQ
RBDSQ-T: RBDSQ total score
REM: Rapid eye movement
ROC: Receiver operating characteristic
SD: Standard deviation
SNUBH: Seoul National University Bundang Hospital
SU: Standard units
VPP: Volume of pineal parenchyma
